# Physiological measurements in social acceptance of self driving technologies

**DOI:** 10.1038/s41598-022-17049-7

**Published:** 2022-08-03

**Authors:** Zsolt Palatinus, Márta Volosin, Eszter Csábi, Emese Hallgató, Edina Hajnal, Miklós Lukovics, Szabolcs Prónay, Tamás Ujházi, Lilla Osztobányi, Balázs Szabó, Tamás Králik, Zoltán Majó-Petri

**Affiliations:** 1 Department of Cognitive and Neuropsychology, Institute of Psychology, University of Szeged, Szeged, Hungary; 2 Institute of Cognitive Neuroscience and Psychology, Research Centre for Natural Sciences, Budapest, Hungary; 3 Faculty of Economics and Business Administration, Department of Economics and Economic Development, University of Szeged, Szeged, Hungary; 4 Faculty of Economics and Business Administration, Institute of Business Studies, University of Szeged, Szeged, Hungary; 5 Mindtech Ltd., Vác, Hungary; 6 Pázmány Péter Catholic University, Budapest, Hungary

**Keywords:** Neuroscience, Psychology, Environmental social sciences

## Abstract

The goal of the present study is to examine the cognitive/affective physiological correlates of passenger travel experience in autonomously driven transportation systems. We investigated the social acceptance and cognitive aspects of self-driving technology by measuring physiological responses in real-world experimental settings using eye-tracking and EEG measures simultaneously on 38 volunteers. A typical test run included human-driven (Human) and Autonomous conditions in the same vehicle, in a safe environment. In the spectrum analysis of the eye-tracking data we found significant differences in the complex patterns of eye movements: the structure of movements of different magnitudes were less variable in the Autonomous drive condition. EEG data revealed less positive affectivity in the Autonomous condition compared to the human-driven condition while arousal did not differ between the two conditions. These preliminary findings reinforced our initial hypothesis that passenger experience in human and machine navigated conditions entail different physiological and psychological correlates, and those differences are accessible using state of the art in-world measurements. These useful dimensions of passenger experience may serve as a source of information both for the improvement and design of self-navigating technology and for market-related concerns.

## Introduction

Autonomous transportation is a field of innovation being rapidly advanced in the past decade. Most major car manufacturers are racing towards mass production of partly or fully autonomous vehicles. Moreover, mobility providers like Uber or Lyft have started their own programs, and tech companies like Google or Nvidia have also entered the field. Despite all the effort that goes into the engineering and the design of self-navigation, there are concerns about the general acceptance of autonomous vehicles. Those concerns include uncertainty about whether the general public would be willing to purchase semi or fully autonomous cars at a rate that justifies mass production, and ethical uncertainty about safety and responsibility, among others. Since the advent of testing self-driving cars in real traffic situations there have been numerous efforts to assess acceptance, anxiety, arousal, and other emotional aspects of this particular new technology^1^. Besides asking people to report their feelings and experiences, a reliable source of information is the tracking of biological measures like heart rate, muscle activity, eye movements, or electroencephalography (EEG) signals. Up until very recently, the non-portability of certain advanced neurological and behavioral techniques such as motion tracking, EEG or electromyography (EMG) often confined these investigations to the laboratory. Instead of real-world passenger experience, researchers often used video recordings, virtual reality (VR) or computer simulations^2–5^. However, recent improvements in measurement technology have made it viable to record these biological signals in the actual vehicles in focus. Our study is aimed at (1) establishing a viable research method for the psychological and physiological measurement of passenger experience in self-navigating vehicles and (2) finding useful dimensions of covariability in the recorded data. To this effect, we selected an initial set of analyses that to our knowledge were likely to uncover cognitive/affective patterns in the EEG and eye-tracking data. The overall aim of our research is to establish a set of measurements that indicate affective engagement in experiencing autonomously driven passenger cars.

Self-driving vehicles are radical innovations which according to our current knowledge will overturn the daily lives and decades-old habits of all people in developed countries—whether they are involved in transport as drivers, cyclists, pedestrians, passengers, etc.^6^. Technological development related to self-driving vehicles has greatly accelerated recently: at present, self-driving vehicle tests are being conducted in nearly 200 cities. Hence, it seems that more developers are in the last phase of technical development and are getting ready to enter production^7^.

With a very few exceptions, developers focus solely on technological development, however, the spread of self-driving vehicles depends not only on the technological development but also on legislative framework, infrastructure, and social acceptance^8^. One of the most important factors regarding innovation diffusion are decisions on innovation: the acceptance or the rejection of innovations and their expansion is based on people’s judgments and decisions^9^. Therefore, development work shall be extended in the direction of mapping social acceptance as accurately as possible, thereby accelerating social adaptation in the interest of the society and its members’ ability to process the projected drastic change. Our research was particularly aimed at finding further sources of information about affective engagement in a real-world situation.

In the last decades of the twentieth century Information Systems (IS) were adopted in both organizational and domestic environments. Behavioral researchers wanted to know which factors influenced the acceptance and use of these new technologies. As a result, several technology acceptance research models were developed. Initially, there were the Theory of Reasoned Action (TRA)^10^, and the Theory of Planned Behavior (TPB)^11^. Davis et al.^12^ merged these theories into the Technology Acceptance Model (TAM). All the above-mentioned models assume that the actual use of a new technology depends on one’s behavioral intention (BI) to apply the technology itself. BI is directly moderated by one’s attitude towards use (A). In the TAM model, Perceived Ease of Use (PEU) and Perceived Usefulness (PU) have a direct aggregated moderating effect on A. In the TAM 2 model, PEU is described by Davis^12^; who later^13^ described PU. Venkatesh et al.^14^ proposed a unification of the existing technology acceptance models and presented the Unified Theory of Acceptance and Use of Technology (UTAUT) adding Social Influence (SI), Facilitating Conditions (FC) and moderating variables (Age, Gender, Experience and Voluntariness) to the model. The UTAUT 2 research model^15^ is designed to allow researchers to investigate consumer acceptance and the use of new technologies, adding new moderating factors (Hedonic Motivation, Price Value, Habit) and removing one (Voluntariness of Use) from the model.

The UTAUT 2 model is widely used to predict technology acceptance and thus future behavior. However, it is an interesting question how well the commonly used questionnaire survey can collect real and valid information. Consider for example the reporting of the subject's own emotional state (called Hedonic motivation in the UTAUT 2 model). Respondents often find it difficult to identify and report their current emotional states. This is especially true when they were asked about their future (expected) emotional states when traveling in a self-driving vehicle. Concurrently, Nordhoff et al.^16^ asked 9118 car drivers from eight European countries using the UTAUT2 model to explain public acceptance of conditionally automated (L3) cars and found that hedonic motivation was identified as the strongest predictor from all UTAUT 2 variables of individuals’ behavioral intention. Their results indicated that individuals who found conditionally automated cars to be fun and enjoyable were more likely to intend to use them. However, the method for measuring hedonic motivation was still the traditional online survey.

On this basis, it is worth considering what alternative data collection methods could be used to explore these emotional motivations. For this purpose, the UTAUT 2 model can be taken as a framework, where variables can be measured with a more holistic approach—for example, to examine emotional responses by neuroscientific methods rather than direct interviewing or by questionnaires based on hypothetical scenarios. Most studies on attitudes and trust towards autonomous technology utilize self-reporting questionnaires (e.g.,^17,18^). This approach has several limitations: first, most participants respond without any prior experience as a passenger or driver of self-driving vehicles. Second, social desirability factors also might bias the results, therefore the objectivity of such data might be questioned. Recording physiological responses like EEG, galvanic skin response (GSR) or eye movements might be a useful method for eliminating such biases, and results of these measurements can be easily compared with self-reported data. Although a growing amount of research attempted to measure passengers’ biological reactions in autonomous vehicles either utilizing EEG (e.g.^3,4^) or eye-tracking methods^19,20^, we are not aware of any studies in which EEG and eye-tracking data were recorded simultaneously. Recognizing that real-world physiological measurements are in their infancy, we set out to explore multiple sources of biologically anchored information on people’s affective experiences.

### Eye movements indicating emotional and cognitive transitions

Muscle movements in perceptual systems including the head, hands, and feet have been established to indicate cognitive and affective states and transitions. Spectral analyses of head movements and eye movements were repeatedly shown to indicate intentional, goal related cognitive transitions such as problem solving, recognition, or comprehension. The general reasoning behind using complexity measures and spectrum analysis on continuous physiological data focuses on the source of cognitive and emotional change. Such changes are likely to be distributed within the complex network of physical, electro-chemical, nervous, muscular, and behavioral interactions. These are fundamental to complex systemic behavior, such as weather patterns, ecological dynamics, stock market fluctuations, or functions of the nervous system. Natural systems often exhibit power-law distributions, and the variation of their components is correlated across both spatial and temporal scales^21,22^. These correlations allow inquiry into the number and strength of interactions between the active components. In recent years, the concept of multifractality has become more popular in modeling, exploration, and prediction of complex dynamical system behavior^23,24^. In psychology, multifractal concepts and tools became available right after the introductory reports (i.e.^25^) and they were followed by tutorials and practical guidance^26,27^.

Differences in the distribution of fractal dimension and the width of the multifractal spectrum have been reported as significant and reliable markers in various cognitive tasks including problem solving^28^, magnitude perception^29,30^, perceptual intent^31^, visual recognition^32^, comprehension^33^, and memory^34^. Recent works more specifically related to the cognitive/affective domain also emphasize useful dimensions in the multifractal spectrum of eye-movements^35–37^. Inspired by these relatively recent findings, our experimental research methods on the eye-tracking data included fractal and multifractal analysis.

### EEG signatures of emotional and cognitive transitions

The human brain is constantly active, resulting in electrical signals that can be measured from the scalp by EEG. The EEG oscillations are mainly classified according to their frequency bands: for example, the delta band is referred to as activation below 4 Hz, the theta band reflects activity between 4 and 8 Hz, the alpha band between 8 and 12 Hz, beta between 13 and 30 Hz, and activity higher than 20 Hz is referred to as the gamma band^38^.

Numerous studies target EEG correlates of emotional and motivational states by differences in alpha band power between the right and left hemispheres^39,40^. Frontal alpha asymmetry is a result of the subtraction of left frontal alpha power from right frontal alpha power after log-transforming the values to normalize distributions (log F4 minus log F3). The result is referred to as relative left or relative right frontal activity: when the result is more positive, relative left activation is present and when the result is more negative, relative right activation is present. High scores therefore indicate more positive or approaching attitudes while lower scores indicate more negative or withdrawal attitudes^40–42^.

When classifying mental states or cognitive processes from relaxed to alerted or stressed states, usually the ratios of higher frequency (beta, gamma) and lower frequency (alpha, theta) powers are compared^3,43^. Lower frequencies are dominating in more relaxed states and sleep while higher frequencies are present in more aroused or stressed states. For example, increased beta/alpha ratio^3,44^, decreased alpha/beta and theta/beta ratio^45^ or increased relative gamma ratio^46^ were found to correlate with stress level. Higher relative gamma was also detected during enhanced attention and concentration^3^.

A growing number of studies recorded EEG while participants were exposed to self-driving technology, mostly by sitting in a simulator^2–5^. For example, Park and colleagues^4^ found an increasing beta-to-alpha power ratio when a participant was passively watching positive scenarios (the car performing smoothly on a highway) and negative scenarios (the same car driving erratically and violating common rules of the road) of self-driving cars, revealing elevated stress levels when the participants were exposed to simulated dangerous situations. In a similar simulator study, the most effective takeover warning signals were accompanied by enhanced ratios of higher frequencies, suggesting enhanced stress, attention, and alertness^3^.

Most recently, Seet et al.^5^ investigated the impact of autonomous vehicle malfunction on human trust by combining EEG and self-reported questionnaires in a simulation environment. During the simulation, participants actively drove the vehicle in an urban environment and from time to time, scenarios with malfunctions (e.g., the vehicle drove at high speed through junctions or even crashed) occurred. In the Conditional Automation Driving phase, participants were able to take over control when needed, while in the Full Automation Driving condition no takeover function was present. Results showed that participants’ preference was significantly higher toward the situation when they were able to take over control. Besides, the frontal alpha power reduction in the right but not in the left hemisphere during malfunctions in the fully automated condition can also be interpreted as an enhanced motivation of the driver towards controlling the vehicle.

Although a compelling advantage of studies utilizing simulators is that they offer high control settings in lab environments, this control comes at the cost of ecological validity^47,48^. Since real-world physiological measurements in autonomous navigation systems is a relatively new area, our hypotheses were outlined carefully. First, we expected our complexity measure—multifractal spectrum width—to be sensitive to differences in the human driver versus autonomous driving conditions. Wider spectra indicate more types of causal relationships shaping the fluctuations of the eye movements. Substantial differences between the two conditions may signal that the autonomous condition is significantly different from the eye movements in the usual passenger experience. Second, we expected to find that the novelty and excitement of the experience would manifest itself in higher levels of valence and arousal in the EEG data. Third, we expected that some personality or anxiety measures in psychological surveys would correlate with physiological measures.

## Methods

### Participants and procedure

For the present study, 38 healthy adults volunteered (mean age: 30.947 years, SD = 7.414 years, 13 females, 4 left-handed). All of them reported normal or corrected-to-normal vision and hearing and no psychiatric or neurological problems. Participants received no monetary compensation and all of them gave written informed consent prior to the study. The experiment was conducted in accordance with the Declaration of Helsinki and the protocol was approved by the United Ethical Review Committee for Research in Psychology (EPKEB), Hungary. EPKEB ref. number 2020-89, approved on 07/06/2020 (http://epkeb.ttk.hu/).

Before the experiment, participants were required to fill personality and demographic questionnaires via an online form. Because the focus of the present study is to measure physiological correlates of those experiencing autonomous drive, we present the details on questionnaire data in the Supplementary Information. The experiment took place at Szeged Airport (ICAO: LHUD), an airport serving Szeged, a city in Csongrád-Csanád county, Hungary. The airport has one asphalt paved runway designated 16R/34L which measures 1185 by 30 m. The runway and a service road leading to the runway were used for the tests. Before the test runs in the vehicle (TESLA Model X) participants were debriefed and both the eye-tracking glasses and the EEG electrodes were mounted. Eye-tracking and EEG data were routed into two separate portable laptop computers. First, a baseline measurement was applied to map participants’ reactions to visual stimuli approximately 60–70 cm distance from the display. Twenty-three pictures with different valence and arousal evoking values were selected from the Open Affective Standardized Images Set (OASIS^49^). Each picture was presented for 5 s, followed by a blank black screen for another 5 s. Participants were instructed to relax and look freely at pictures without any additional task. Baseline measurement lasted about 4 min.

**Figure 1:**
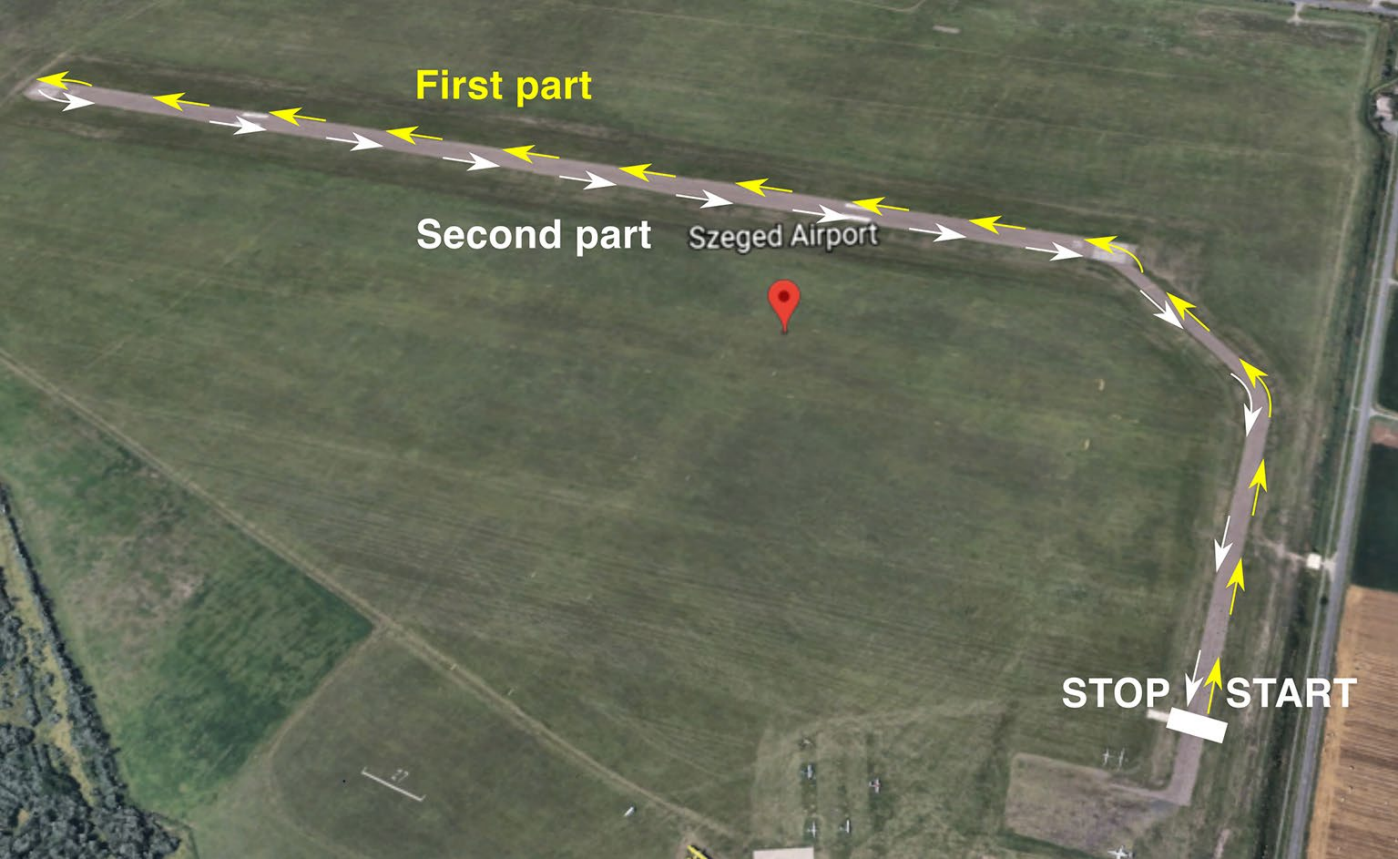
Birdseye view of the runway and the service road used in the experiment. Yellow arrows indicate the outbound path towards the end of the runway (First part), white arrows indicate the inbound path back to the starting position (Second part). Source: Google Earth (n.d.). [Google Earth map of Szeged Airport]. Retrieved June 10, 2022, from https://earth.google.com/web/@46.24993219,20.09495634,79.94030556a,1403.84271768d,35y,-117.93221529h,45.00445248t,0r.

After the baseline measurement, the participant, the driver and two experimenters with the recording computers entered the vehicle. The driver was seated in the driver’s seat (front left) and the participant was seated in the passenger’s seat (front right) while the two experimenters were seated in the back seat operating the recording equipment. The participant was instructed to relax and behave like a passenger in general while minimizing movement. The route was taken twice: first, a professional driver was driving (*Human condition*), and in the second round, the self-driving mode was switched on and the driver released the steering wheel (*Autonomous condition*). The bird-eye view of the route used in the experiment is presented in Fig. 1. Both types of blocks lasted about 2 to 3 min and were recorded separately. Before and after the two runs, participants filled out the Positive and Negative Affect Scale (PANAS).

### Measures and data analyses

#### Eye tracking recording

For eye tracking we used the Core System manufactured by Pupil Labs (Berlin, Germany) attached to a portable laptop computer. This system has two cameras: one is positioned forward to record the field of view of the wearer in HD video at 30 Hz, and a second infrared camera recorded the participant’s eye movements. Pupil Player software developed by the manufacturer (version 3.3, https://pupil-labs.com/products/core/), was used to determine the movement of the eyes in 3D.

##### Preprocessing data

Th**e** raw data of the time series of the *xy* pupil positions were transcoded into a single one-dimensional vector, indicating the intensity of the movements without regard to movement directionality. Data recorded at each trial were divided into a first half of the run (before the car took a U turn at the end of the runway) and a second half (after the U turn) (see Fig. 1).

##### Data analysis

Multifractal analysis of each half run was applied using the Chhabra and Jensen (CJ) method^50^. This is a canonical “direct” algorithm for calculating the multifractal-spectrum width that samples measurement series at progressively larger scales. Empirical examples suggest that the CJ method is suitable for assessing biological movements similar to the movement of the eye. To assess participants’ reactions before and after the U turn in the Human and Autonomous conditions, Condition (Human/Autonomous) × Part (First/Second) repeated measures ANOVA was applied.

#### EEG recording

Continuous EEG data was recorded at a 200 Hz sampling rate with a portable OpenBCI 4-channel Ganglion board utilizing Lab Streaming Layer (LSL) from OpenBCI GUI. Four gold cup electrodes were attached to participants’ heads with conductive paste (Ten20) in accordance with the 10–20 system^51^ to F3, F4, FPz and Oz. Two additional electrodes were attached to the left and right mastoids serving as reference and ground electrodes, respectively. Impedances were kept below 30 kΩ.

##### Preprocessing data

The continuous EEG was filtered offline; a bandpass filter (7–48 Hz, 9th order Butterworth) was applied to the data as this particular range characterized the frequencies of our interest. After filtering the whole blocks, continuous data were segmented into 4 s long epochs with 2 s overlapping parts. Epochs with a signal range exceeding ± 100 µV (typically due to movement or blink artefacts) were excluded from further analysis. Power spectral densities (PSD) were calculated to alpha (8–12 Hz), beta (13–30 Hz) and gamma (30–45 Hz) band ranges utilizing the Welch method. For each epoch, an index for valence (affectivity) and arousal were computed. Affectivity corresponded to frontal alpha asymmetry and was calculated as the difference between log_10_ transformed values of F4 and F3 with higher values representing more positive emotional valence^41^. As higher frequencies were found to index a more aroused state in frontal areas^3,45^, arousal was defined as the ratio of PSD in the beta and gamma range to the alpha range at the averaged F3 and F4 electrodes. Both for affectivity and arousal, outlier epochs which deviated from the participants’ mean at least by 3 SD were removed before averaging data. As with the eye-tracking data, the first and second halves of each condition were also averaged separately and submitted to statistical analysis as described later.

##### Statistical analyses

Similar to eye tracking data, differences between the first and second halves of the route were compared in the two conditions by Condition (Human/Autonomous) × Part (First/Second) ANOVAs separately for frontal alpha asymmetry (affectivity) and arousal values. Interactions were followed up with paired t-tests. Statistical analyses were conducted in R (version 4.0.5^52^). Generalized eta-square (η^2^_G_) effect sizes^53,54^ and statistical power^55^ are also reported.

## Results

### Eye tracking results

Multifractal spectra of one-dimensional eye displacement data were calculated for the first and second halves of each trial (Part). Out of the 38 participants, in five cases our equipment produced suboptimal data quality with less than 0.800 mean confidence level in capturing the real position of the pupil (where 1 designates maximum confidence). These five cases were excluded from further eye-tracking based data analyses. Differences were compared for Part (First/Second) and Condition (Human/Autonomous) in a repeated measures ANOVA, showing a significant Condition main effect: (F(1, 32) = 40.816, p < 0.001, η^2^_G_ = 0.205, power = 96%), suggesting a narrower MF spectrum when the car was driving in autonomous mode. No other effects were significant.

### EEG results

**Figure 2:**
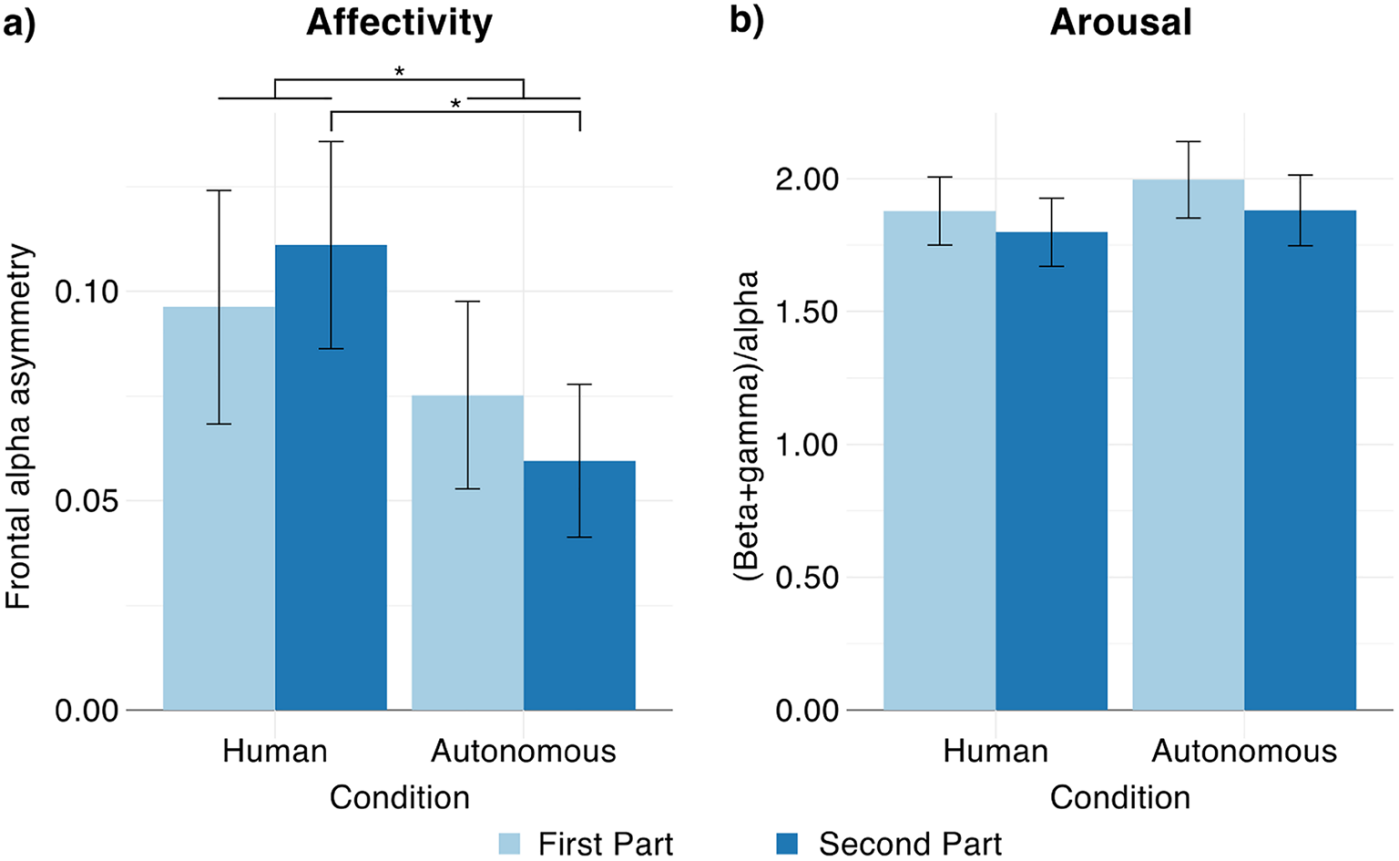
EEG correlates of passengers’ experience in the autonomous vehicle. Light blue bars denote the first part and dark blue bars denote the second part of the ride in the Human and Autonomous condition. Participants were characterized with frontal alpha asymmetry (**a**) and arousal (**b**) scores. Whiskers indicate the standard error of the mean; star sign denotes significant (p < 0.05) differences.

Affectivity values are presented in Fig. 2a while arousal values are presented in Fig. 2b. Because of the extensive amount of movement artefacts (more than 60% of the segments), and because of technical problems during the data acquisition (lost connection between the OpenBCI board and computer during the ride), data of 7 persons were excluded from EEG analysis. That is, EEG analysis was performed on data of 31 participants (22 males, 9 females, mean age = 31.258 years, SD = 7.806, range: 22–57 years).

#### Affectivity (frontal alpha asymmetry)

The Condition × Part ANOVA showed a significant Condition main effect: (F(1, 30) = 9.871, p = 0.004, η^2^_G_ = 0.019, power = 100%), suggesting larger avoidance, and less positive reactions when the car was driving in autonomous mode. Furthermore, the significant Condition × Part interaction was significant as well: F(1, 30) = 6.292, p = 0.018, η^2^_G_ < 0.001, power = 54%). The follow-up t-tests indicated that while the first part of the ride was similar in both conditions (t(30) = 1.692, p = 0.101, power = 56%), participants’ reactions were more negative after the U turn in the Autonomous condition (t(30) = 3.780, p < 0.001, power = 100%). The Part main effect was not significant (F(1, 30) = 0.003, p = 0.958, η^2^_G_ < 0.001, power = 5%).

#### Arousal

The Condition × Part ANOVA showed no significant differences, suggesting that arousal was similar in the two conditions (Condition main effect: F(1, 30) = 3.295, p = 0.080, η^2^_G_ = 0.005, power = 83%) and during the two parts of the ride (Part main effect: F(1, 30) = 3.834, p = 0.059, η^2^_G_ = 0.004, power = 46%). The Condition × Part interaction was not significant either: F(1, 30) = 0.217, p = 0.066, η^2^_G_ < 0.001, power = 7%.

## Discussion

The present study aimed to investigate people’s experiences and feelings while being a passenger of an autonomous vehicle as indexed by MF power and EEG spectral density patterns and by self-reported questionnaires. We found an overall narrower MF spectrum width in the Autonomous condition. Regarding EEG, frontal alpha asymmetry values were higher in the Human condition compared to the Autonomous condition, while arousal levels did not differ between the two conditions.

Eye-tracking data indicated an overall narrower MF spectrum width in the Autonomous condition. Beyond the obvious usefulness of a seemingly reliable biological index of the travelling conditions the direction of this difference holds further questions. Narrower spectra are often characterized as indicative of fewer governing forces in the dynamical system. Arguably, the movement structure of the car in self-driving condition could be governed by fewer degrees of freedom compared to the neuro-muscular system of a human driver. Alternatively, heightened alertness in the unfamiliar autonomous condition may have resulted in lower variability in eye movements. This strong effect may even be related to both factors at the same time. More testing is required to identify the discrepancy, which in turn may possibly provide a meaningful measure in the future.

The significantly higher frontal alpha asymmetry values in the Human than in the Autonomous condition suggest that participants’ affect was less positive in the autonomous mode. Despite the scarcity of literature on frontal alpha asymmetry related to self-driving vehicles, this pattern is comparable with results of Abdur-Rahim et al.^2^ who found enhanced right frontal activation when participants drove autonomous wheelchairs through a narrow path. However, in their study^2^, in the self-driving mode EEG signals were insufficient to predict stress but GSR signals indicated higher stress levels, probably due the lack of control over the vehicle. In contrast, Seet et al.^5^ demonstrated decreased alpha power in the right frontal areas when participants had no option to take over the drive of an autonomous car which suggests that participants felt motivated to control the driving. They also speculated that frontal alpha activity might be a neural correlate of trust in autonomous vehicles as it was modulated by the urge to take over control of the vehicle. Even though no dangerous situations were indicated in the present study, participants were not able to control the car either. Therefore, observing the lack of control over the vehicle in the Autonomous condition could lead to lower alpha asymmetry scores, implying lower trust and less positive feelings, which was more pronounced in the second part of the ride. This is in line both with survey and behavioral results in the literature. Several survey studies suggest that societies are neutral or even resistant toward AV in general^56–60^. Regarding behavioral results, Xu et al.^1^ found that directly experiencing autonomous technology increased trust, but decreased interest in fully automated driving technology. The authors explained these results with subject expectations being too high due to media hype, or with imperfect technology^1^. The role of imperfect technology was supported by further results: exposing participants to AV malfunctions as a passenger at a test track led to significant decrease in trust and attitude and increased negative affect^48^. In the present study, the vehicle was unable to perform every maneuver (for example full turnabouts) either. According to subjects’ reports it did not drive as smoothly as with a human driver, which could contribute to more negative affect in the Autonomous condition. There is evidence that different driving styles and human driver conditions are detectable in the movement patterns of the vehicle^61,62^. This may provide another possibility to improve user experience in autonomous vehicles by fine tuning their steering, accelerating and decelerating patterns to the point that it resembles human driving more closely, without sacrificing any benefits^63^.

In contrast with frontal alpha asymmetry, and unsurprisingly, a high arousal level was retained during the whole experiment. This conforms to the literature as well: despite the entirely safe and predictable rides, being a passenger in an autonomous car mounted with EEG and an eye-tracker could be an exciting experience either in a positive or negative way, leading to constantly enhanced arousal and alertness^3,44,47^.

Although the present study was launched as a pilot project, it has numerous strengths. We measured EEG and eye movements simultaneously, which combined with self-report evaluations allowed us to reveal relationships between these three methods. In contrast to the majority of studies applying VR or other simulation settings^2–5^, the present study was carried out in a real-world environment in an autonomous vehicle. Though measurements could not be conducted in traffic due to safety reasons, simply experiencing the ride can be evaluated as ecologically valid.

There are several limitations of the present study: Firstly, most of our analyses can be regarded as explorational, especially the correlations between biological signals and personality traits (see Supplementary Information)—suitable for a pilot study but more systematic and hypothesis-driven testing is needed for more reliable results. Secondly, to keep gradual change from the baseline to Human and Autonomous modes, the order of the conditions was the same for all participants. This order effect can explain for example the decreasing frontal alpha asymmetry to the Autonomous condition: the motivation or the positive affectivity which was present at the beginning could attenuate to the end of session, especially when participants’ expectations of the autonomous technology were not met. In the future, at least the order of the Human and Autonomous conditions should be balanced. A third weakness was that participants were highly motivated volunteers, which could significantly impact both their neural responses and attitudes.

In summary, the present study demonstrated differences in eye-movement patterns and neural correlates between experiencing human- and self-driven modes of autonomous vehicles. Our preliminary findings suggest that although participants’ affectivity was slightly less positive in the autonomous mode, they were more aroused, which is also compatible with personality traits and subjective reports. With respect to the UTAUT2 model, our results clearly indicated that neuropsychological and behavioral data collection and analysis may indeed enhance the predictive power and usefulness of traditional data collection methods especially in the affective domain. Furthermore, it also might contribute to the debate on the presence of a general acceptance factor (GAF) of technology^64,65^. Future studies need to investigate the exact relationship between different factors more systematically and ideally in circumstances that are, within safety measures, even more comparable with real life driving scenarios. The differences in neural signatures might be useful and promising for exploring neural correlates of trust and acceptance of autonomous vehicles.

## Supplementary Information

Supplementary Information.

## Data Availability

The datasets generated during and/or analyzed during the current study are available in the OSF repository, (https://osf.io/dhcaf/?view_only=a12e150a53a94585a32597c129ee5d4f). Codes used in the analyses of the datasets are available from the corresponding author on reasonable request.
