# Supplementary material for: Physiological measurements in social acceptance of self driving technologies
Source: Sci Rep. 2022 Aug 3;12:13312. doi: 10.1038/s41598-022-17049-7 (PMC9349214; doi:10.1038/s41598-022-17049-7)
Supplement: Supplementary file 1 — Supplementary Information. [file 41598_2022_17049_MOESM1_ESM.docx]

**Supplementary Information**

**Introduction**

Despite of the rapid pace of the technological innovations, the most dominant barriers in adaptation of autonomous vehicles seem to be rather psychological than technical [1]. Although a growing number of studies aim to investigate personality traits (e.g. [2-4]) contributing to attitudes toward AV, the results are not equivocal. One of the main reasons for the controversial results might be that the vast majority of the respondents have never experienced being a passenger in an autonomous vehicle. Therefore, as an exploratory part of our study, we aimed to map which personality factors might be related to the with physiological correlates of experiencing autonomous mode.

***Big 5 factors:*** Higher neuroticism (that is, lower level of emotional stability) negatively impacted the predicted usefulness of new technologies [5], and drivers with higher level of neuroticism were found to be more concerned about the data security issues as well [6] and showed lower acceptance of AV technology [4]. On the other hand, agreeability was related to higher willingness to share personal data [6], to have a more positive perspective on the benefits of AV [4]. Individuals with lower level of extraversion [7] and with higher level of conscientiousness and openness require higher level of control and they need of more information before using an autonomous vehicle [2], leading to lower trust as measured in a driving simulation task [3]. In contrast, other studies found that higher level of openness was associated with more willingness for giving up the control over the vehicle [7] and higher acceptance of AVs [4].

***Sensory sensation seeking:*** Higher level of sensory sensation seeking is associated with enhanced risk taking, which is linked to drive at higher speed and to keep shorter headways between vehicles [8, 9]. It was demonstrated that individuals with higher level of sensory sensation seeking were more open to use driving assistance applications [10] and to adopt autonomous vehicles [7]. On the other hand, Cho and Ji [11] did not find any association between sensory sensation seeking and adaptation of AVs, probably because driving an autonomous vehicle is not a novel enough experience.

***Locus of control:*** External control was found to be associated with higher acceptance of AV: individuals who agreed that safe drive depends on external factors instead of the driving skills of the driver were more open to use AV [11]. Contrary, locus of control was not associated with the attitude toward AV but it affected the driving style [9].

**Results**

Regarding MF spectrum, ego-resilience was in a significant positive relationship with the MF spectrum width in the Autonomous condition (r_s_(33) = 0.36, p = 0.04, power = 63.640%) showing that more ego resilience was related to wider spectra in the unfamiliar condition.

Affectivity correlated positively with **PANAS positive scale before the ride** both in the Human (r_s_(29) = 0.405, p = 0.024, power = 51.548%) and in the Autonomous conditions (r_s_(29) = 0.519, p = 0.003, power = 52.842%). No other effects were significant (all r_s_ values < 0.350, all p values > 0.053).

Arousal values in the Autonomous condition showed significant positive correlations with **Neuroticism** (r_s_(29) = 0.379, p = 0.035, power = 51.476%) and **Agreeableness** (r_s_(29) = 0.378, p = 0.036, power = 51.355%) factors, and positive relationship with general **attitude towards AV** (r_s_(29) = 0.394, p = 0.028, power = 51.215%). No other effects were significant (all r_s_ values < 0.332, all p values > 0.068).

**Discussion**

The relationship between MF and EEG power spectrum and personality traits can be regarded as exploratory findings, therefore they need to be interpreted cautiously, especially when considering low statistical power. The psychological survey data had some notable co-variation with the MF spectrum of the eye movements. Ego resilience showed positive correlation with the measured MF spectrum in the *Autonomous* condition. This finding suggests that people with higher levels of ego resilience were not affected as greatly by the difference in the two conditions. This finding further indicates that our measures may serve as sources of useful information for future design and marketing purposes.

Affectivity in both conditions correlated positively with the positive affect before the ride, indicating that those who were in a more positive mood before experiencing AV, they exhibited higher frontal alpha asymmetry values. This suggests that more positive mood was associated with more positive affect in the AV.

Arousal values correlated with Agreeableness scores suggesting that participants who were more friendly and empathetic exhibited higher arousal in both conditions. The positive correlation between neuroticism and arousal measured in Auto condition implies higher arousal levels in more neurotic individuals. This result is in line with literature on the positive relationship between higher frequency oscillations and neuroticism [12-14]. Furthermore, the positive relationship between the arousal level in the Autonomous condition and acceptance of autonomous vehicles might indicate that participants with higher acceptance exhibited more excitement when the vehicle was in autonomous mode.

Again, such correlations can easily lead to unproven speculations about self-rated traits and EEG oscillatory activity and need to be tested on larger sample [15-16].

**Measures and data analysis**

Participants filled an online questionnaire before the experiment which contained question about demography and the following personality scales. Spearman correlations were calculated between the scores of the scales and physiological data (multifractal spectrum, affectivity, arousal).

***Ego-Resiliency Questionnaire (ER89).*** We used the Hungarian version of the Ego-Resiliency Scale (ER89) including three factors utilizing a 4-point Likert scale: 1) Active engagement with the world, 2) Integrated performance under stress, and 3) Repertoire of problem-solving strategies [17]. Higher scores indicate better problem-solving strategies, better performance under stress, and more active engagement with the world, respectively.

***Positive and Negative Affect Schedule (PANAS).*** The Hungarian version of PANAS was utilized [18]. Negative or positive statements are rated on a 5-point Likert scale. Higher scores represent a higher level of positive or negative affective response. We administered the questionnaire before and after the ride in the AV.

***Brief 30-item Bipolar Rating Scale for the Five Factor Model of Personality (BBRS-30).*** We used the Brief 30-item Bipolar Rating Scale assessing the following personality traits by a 7-point semantic differential scale: Extraversion, Neuroticism, Conscientiousness, Agreeableness, Openness [19]. Higher scores indicate higher levels of the relevant traits.

***Brief Sensation Seeking Scale (SSS).*** We used the Hungarian version, in which each item contains two choices and the responders have to choose which of the options describes themselves better. Higher scores indicate a higher level of sensation seeking [20, 21].

***Spielberger State-Trait Anxiety Inventory* *(STAI).*** We used the Hungarian version of the self-report State-Trait Anxiety Inventory. The responders indicate on a 4-point Likert rating scale the frequency at which they experience specific anxiety symptoms at a particular time (state anxiety) or in general (trait anxiety). Higher scores indicate higher anxiety [22].

***Rotter’s Locus of Control Scale***. According to the theory on locus of control [23], people with an internal locus of control believe that the outcomes of their actions are results of their abilities, while people with external locus believe that life events are out of their control and are results of external factors (e.g. fate or luck). We used the Hungarian adaptation consisting of 29 items, and the participants had to select between two statements for each question that they agreed with the most. Lower scores indicate internal control while higher scores indicate external control.

***Trolley Dilemma.*** The trolley problem is a classic series of thought experiments asking about sacrificing a person in order to save several others. Thirteen different trolley dilemma scenarios were created utilizing moralmachine.net (e. g. [1]) and responders had to decide what the self-driving car should do. Here we calculated the percentage of “save passengers'” (i.e. “sacrifice pedestrians”) answers for all of the participants.

***Attitudes towards autonomous vehicles.*** To assess participants’ attitudes towards autonomous vehicles, we translated items of the questionnaire utilized by Charness et al. [7] to Hungarian. Some of the questions were asked in the form of a 5-point Likert scale, while other questions could be answered on a 10-point Likert scale. We converted responses on the 5-point Likert scale questions (from 1-5) to be interpretable on a 10-point Likert scale. That is, 1 corresponded to 1, 2 corresponded to 3.25, 3 corresponded to 5.5, 4 corresponded to 7.75 and 10 corresponded to 10. Items 4, 6, 7, 9, 10, 12, 13, 14, 15 and 16 were reversed in order to that higher scores indicate higher acceptance. To get a single score for each participant, we calculated the mean score of their answers. Higher values indicate higher acceptance of self-driving vehicles and indexing greater trust in them.

**References**

1. Shariff, A., Bonnefon, J.-F. & Rahwan, I. Psychological roadblocks to the adoption of self-driving vehicles. *Nature Human Behaviour* **1**, 694–696 (2017).
2. Amichai-Hamburger, Y., Mor, Y., Wellingstein, T., Landesman, T. & Ophir, Y. The Personal Autonomous Car: Personality and the Driverless Car. *Cyberpsychology, Behavior, and Social Networking* **23**, 242–245 (2020).
3. Li, W. *et al.* Personality Openness Predicts Driver Trust in Automated Driving. *Automot. Innov.* **3**, 3–13 (2020).
4. Qu, W., Sun, H. & Ge, Y. The effects of trait anxiety and the big five personality traits on self-driving car acceptance. *Transportation* **48**, 2663–2679 (2021).
5. Devaraj, S., Easley, R. F. & Crant, J. M. **Research Note** —How Does Personality Matter? Relating the Five-Factor Model to Technology Acceptance and Use. *Information Systems Research* **19**, 93–105 (2008).
6. Kyriakidis, M., Happee, R. & de Winter, J. C. F. Public opinion on automated driving: Results of an international questionnaire among 5000 respondents. *Transportation Research Part F: Traffic Psychology and Behaviour* **32**, 127–140 (2015).
7. Charness, N., Yoon, J. S., Souders, D., Stothart, C. & Yehnert, C. Predictors of Attitudes Toward Autonomous Vehicles: The Roles of Age, Gender, Prior Knowledge, and Personality. *Front. Psychol.* **9**, 2589 (2018).
8. Hoedemaeker, M. & Brookhuis, K. A. Behavioural adaptation to driving with an adaptive cruise control (ACC). *Transportation Research Part F: Traffic Psychology and Behaviour* **1**, 95–106 (1998).
9. Payre, W., Cestac, J. & Delhomme, P. Intention to use a fully automated car: Attitudes and a priori acceptability. *Transportation Research Part F: Traffic Psychology and Behaviour* **27**, 252–263 (2014).
10. Rudin-Brown, C. M. & Noy, Y. I. Investigation of Behavioral Adaptation to Lane Departure Warnings. *Transportation Research Record* **1803**, 30–37 (2002).
11. Choi, J. K. & Ji, Y. G. Investigating the Importance of Trust on Adopting an Autonomous Vehicle. *International Journal of Human-Computer Interaction* **31**, 692–702 (2015).
12. Farahi, S., Asghari, M., Gorji, A., Bigdeli, I. & Moshirian Farahi, S. M. M. Cortical brain activities related to neuroticism and extraversion in adolescence. *Neuropsychological Trends* 39–60 (2019).
13. Moshirian Farahi, S. M., Asghari Ebrahimabad, M. J., Gorji, A., Bigdeli, I. & Moshirian Farahi, S. M. M. Neuroticism and frontal EEG asymmetry correlated with dynamic facial emotional processing in adolescents. *Front. Psychol.* **10**, (2019).
14. Webb, C. A. *et al.* Neural Correlates of Three Promising Endophenotypes of Depression: Evidence from the EMBARC Study. *Neuropsychopharmacology* **41**, 454–463 (2016).
15. Korjus, K. *et al.* Personality cannot be predicted from the power of resting state EEG. *Front. Hum. Neurosci.* **9**, (2015).
16. Martínez-Tejada, L. A., Maruyama, Y., Yoshimura, N. & Koike, Y. Analysis of Personality and EEG Features in Emotion Recognition Using Machine Learning Techniques to Classify Arousal and Valence Labels. *Machine Learning and Knowledge Extraction* **2**, 99–124 (2020).
17. Farkas, D. & Orosz, G. Ego-Resiliency Reloaded: A Three-Component Model of General Resiliency. *PLOS ONE* **10**, e0120883 (2015).
18. Gyollai, A., Simor, P., Köteles, F. & Demetrovics, Z. Psychometric properties of the Hungarian version of the original and the short form of the Positive and Negative Affect Schedule (PANAS). *Official Journal of the Hungarian Association of Psychopharmacology*
19. Shafer, A. B. Factor analyses of Big Five Markers with the Comrey Personality Scales and the Howarth Personality Tests. *Personality and Individual Differences* **26**, 857–872 (1999).
20. Andó, B. *et al.* The 7-Item Brief Sensation Seeking Scale (SSS-7-HU). *Journal of Mental Health and Psychosomatics* **10**, 139–152 (2009).
21. Zuckerman, M. *Behavioral Expressions and Biosocial Bases of Sensation Seeking*. (Cambridge University Press, 1994).
22. Sipos, K. & Sipos, M. The development and validation of the Hungarian Form of the State-Trait Anxiety Inventory. *Series in Clinical & Community Psychology: Stress & Anxiety* **2**, 27–39 (1983).
23. Rotter, J. B. Generalized expectancies for internal versus external control of reinforcement. *Psychological Monographs: General and Applied* **80**, 1–28 (1966).
